# Characterization of antibody‐dependent cellular phagocytosis in patients infected with hepatitis C virus with different clinical outcomes

**DOI:** 10.1002/jmv.29381

**Published:** 2024-01-18

**Authors:** Anurag Adhikari, Arunasingam Abayasingam, Nicholas A. Brasher, Ha Na Kim, Megan Lord, David Agapiou, Lisa Maher, Chaturaka Rodrigo, Andrew R. Lloyd, Rowena A. Bull, Nicodemus Tedla

**Affiliations:** ^1^ School of Biomedical Sciences, Faculty of Medicine UNSW Australia Sydney New South Wales Australia; ^2^ Department of Infection and Immunology Kathmandu Research Institute for Biological Sciences Lalitpur Nepal; ^3^ Molecular Surface Interaction Laboratory, Mark Wainwright Analytical Centre UNSW Sydney Sydney New South Wales Australia; ^4^ Graduate School of Biomedical Engineering, Faculty of Engineering UNSW Sydney Sydney New South Wales Australia; ^5^ The Kirby Institute UNSW Australia Sydney New South Wales Australia

**Keywords:** affinity, epitope mapping, hepatitis C, neutralization, phagocytosis

## Abstract

Early neutralizing antibodies against hepatitis C virus (HCV) and CD8 + T cell effector responses can lead to viral clearance. However, these functions alone are not sufficient to protect patients against HCV infection, thus undefined additional antiviral immune mechanisms are required. In recent years, Fc‐receptor‐dependent antibody effector functions, particularly, antibody‐dependent cellular phagocytosis (ADCP) were shown to offer immune protection against several RNA viruses. However, its development and clinical role in patients with HCV infection remain unknown. In this study, we found that patients with chronic GT1a or GT3a HCV infection had significantly higher concentrations of anti‐envelope 2 (E2) antibodies, predominantly IgG1 subclass, than patients that cleared the viruses while the latter had antibodies with higher affinities. 97% of the patients with HCV had measurable ADCP of whom patients with chronic disease showed significantly higher ADCP than those who naturally cleared the virus. Epitope mapping studies showed that patients with antibodies that target antigenic domains on the HCV E2 protein that are known to associate with neutralization function are also strongly associated with ADCP, suggesting antibodies with overlapping/dual functions. Correlation studies showed that ADCP significantly correlated with plasma anti‐E2 antibody levels and neutralization function regardless of clinical outcome and genotype of infecting virus, while a significant correlation between ADCP and affinity was only evident in patients that cleared the virus. These results suggest ADCP was mostly driven by antibody titer in patients with chronic disease while maintained in clearers due to the quality (affinity) of their anti‐E2 antibodies despite having lower antibody titers.

## 1 INTRODUCTION

Hepatitis C virus (HCV) infection is the major cause of severe liver disease, including chronic hepatitis, cirrhosis, and hepatocellular carcinoma. Globally an estimated 71 million people are currently infected with the virus with over 400 000 deaths from chronic HCV‐associated diseases every year.^1^ Although current treatment strategies using direct‐acting antiviral drugs are effective in eliminating the virus, they are expensive and inaccessible for much of the affected population. Thus, the development of a prophylactic vaccine is required to meet the 2030 World Health Organisation target of the global elimination of HCV.^2^ However, due to incomplete knowledge of all the determinants of immunological protection during natural infection and the divergent and highly mutable nature of the virus, the development of an effective vaccine has been challenging. To date, several studies have characterized the early neutralizing antibody response against HCV, along with the early CD4 + T cell help that is associated with HCV clearance,^3^, ^4^, ^5^ whereas, the presence of autoreactive IgM,^6^ late activation of T follicular helper cells,^7^ and increase in exhausted CD4 + T cells phenotype^8^ have been reported to be associated with chronic HCV infection. Moreover, studies showed that viral envelope 2 (E2)‐specific memory B cells undergo earlier expansion among individuals that clear the virus than patients that develop chronic disease.^7^, ^9^, ^10^

However, antibody‐mediated neutralization^11^, ^12^ or early CD4 + T cell functions^13^ alone are not sufficient to protect against HCV infection, thus undefined immunological processes acting independently or in synergy with the beforementioned mechanisms are required for sustained and broad immune protection. In recent years, Fc‐receptor‐dependent antibody effector functions, including antibody‐dependent cellular phagocytosis (ADCP), antibody‐dependent cellular cytotoxicity (ADCC), antibody‐dependent complement deposition (ADCD), and antibody‐dependent respiratory burst (ADRB) have been recognized to play key roles in antiviral immunity.^14^, ^15^, ^16^, ^17^ Among these effector functions, ADCP has been shown to correlate with immune protection against several RNA viruses, including HIV,^18^ ebola,^19^ dengue,^20^ and influenza.^21^ Importantly, in patients with HIV infection, antibodies that neutralize broader HIV variants^22^, ^23^ also possess potent Fc receptor‐dependent effector functions, including ADCP,^24^, ^25^ and antibodies that maintain broadly neutralizing function at later stages of the disease showed an early and stronger Fc‐receptor‐dependent effector function.^14^, ^17^, ^26^, ^27^ A positive relationship between neutralization and ADCP was also observed in patients with severe acute respiratory syndrome coronavirus 2 (SARS‐CoV‐2)^15^, ^28^ and influenza virus^29^ infections. However, the development, and role of ADCP in patients with HCV infection remain unknown. Here we propose that patients with HCV infection mount ADCP that may play a critical role in disease pathogenesis and clinical outcome.

In this study of 67 individuals with HCV infection, we found that patients with chronic HCV infection had significantly higher concentrations of anti‐envelope 2 (E2) antibodies, predominantly IgG1 subclass, than patients that cleared the viruses while the latter had antibodies with better affinities. Importantly, 97% of the patients had measurable ADCP of whom patients with chronic disease showed significantly higher ADCP than those who naturally cleared either GT1a or GT3a viruses independent of sex and age. Epitope mapping indicated that patients with antibodies that target antigenic domains on E2 protein that are associated with neutralization function were also associated with ADCP, suggesting antibodies with overlapping/dual functions. Correlation studies showed that ADCP significantly correlated with plasma anti‐E2 antibody levels and neutralization function regardless of clinical outcome and genotype of the infecting virus. Interestingly, a significant positive correlation between ADCP and affinity was only evident in clearers suggesting that ADCP function is maintained in clearers partly due to the quality (affinity) of their anti‐E2 antibodies despite generally having lower antibody titers.

## 2 RESULTS

### 2.1 Study cohort

The median ages of the patients with HCV infection and those of healthy controls were 41 years (range 34–66 years) and 40 years (range 24–53), respectively, and the male‐to‐female ratios were 1.6:1 and 1.1:1, respectively. 70.1% (47/67) of the patients were infected with a GT1a virus, and 29.9% (20/67) were infected with a GT3a virus (Table 1). The median age of the patients infected with the GT1a virus (38 years) was comparable to those infected with GT3a (41 years), however, the ratios of males to females in patients infected with the GT1a (1.8:1) was higher than the 1.1:1 ratio in those infected with GT3a.

**Table 1:** Demographic profile of the patients with HCV infection.

Parameters	Genotype 1a			Genotype 3a			GT1a & GT3a
Disease outcome	Chronic	Clearer	Total	Chronic	Clearer	Total	Total
Number of participants	24	23	47	12	8	20	**67**
Median days postinfection (DPI)	129.3 (16.5–196)	117.5 (29.6–200)	64.1 (16.5–200)	127.2 (47–250)	108 (12–181.5)	120.5 (12–250)	**70.5 (12–250)**
Median age, years (range)	38 (35–63)	34 (34–66)	38 (35–66)	42 (35–63)	40 (40–47)	41 (35–63)	**41 (34–66)**
Total male participants	16	14	30	7	5	12	**42**
Total female participants	8	9	17	5	3	8	**25**
Male to female ratio	2:1	1.4:1	1.7:1	1.4:1	1.6:1	1.5:1	**1.6:1**

*Note*: Day of infection for each individual patient is defined as the first day of positive results for anti‐HCV IgM antibody by ELISA, and HCV RNA by real‐time PCR is reported and days postinfection (DPI) is then counted from this point on.

Abbreviations: ELISA, enzyme‐linked immunosorbent assay; HCV, hepatitis C virus.

Clinically, 51.1% (*n* = 24/47) of the patients with the GT1a infection and 60% (*n* = 12/20) of those infected with the GT3a virus had a chronic disease. The patients who cleared the GT1a or GT3a virus were marginally younger with a median age of 34 years, than the 38 years for those who developed chronic diseases (Table 1). For those infected with the GT1a, males with chronic disease were twice more than females, however, this is likely a reflection of their overrepresentation in the cohort.

The plasma or sera used in this study were collected at 16–200 days postinfection (DPI) with a median of 64.1 DPI for those infected with the GT1a virus and a median collection time of 120.5 DPI (range 12–250) for patients infected with GT3a virus (Table 1). Within the GT1a‐infected patients, the median DPI in patients with chronic disease was 69.1 (range 16–196) and for those who cleared the virus was 74.3 (range 30–200). Interestingly, the median DPI for patients infected with GT3a was measurably longer with 127.3 days (range 47–250) for those with chronic disease and 108 days (range 12–182) for those who cleared the virus.

### 2.2 Patients with chronic HCV infection had higher anti‐E2 antibody concentrations but lower affinity than those who cleared the viruses

**Figure 1:**
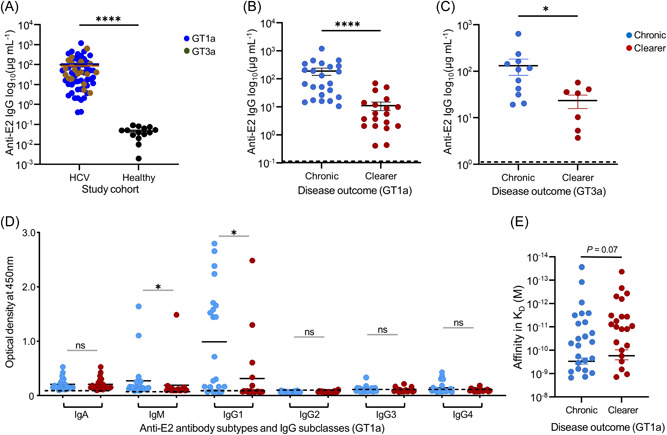
Differences in plasma anti‐E2 IgG levels, antibody types, IgG subclasses, and affinity. ELISA of plasma showed high levels of anti‐E2 IgG in patients infected with GT1a or GT3a HCV than in healthy controls (*p* = 0.0001) (A). Patients with chronic HCV infection had significantly higher levels of anti‐E2 IgG than those who cleared the GT1a virus (B; *p* = 0.0001) or GT3a virus (C; *p* = 0.02). Further analysis of the anti‐E2 antibody types and IgG subclasses produced in response to GT1a HCV infection showed that anti‐E2 IgG1 was the most abundant subclass with those with the chronic disease having significantly more than the clearers (*p* = 0.01), however, there was little detectable IgG2, IgG3, and IgG4 in both patient groups. Anti‐E2 IgA and IgM were also detected in most patients with the latter showing significant differences between chronic and clearers (*p* = 0.01) (dotted lines indicate average value from 15 healthy controls+3 SD) (D). Monitoring of the interaction of plasma anti‐E2 antibodies with E2 protein using SPR showed that on average antibodies from patients that cleared the virus had two times stronger interaction with E2 protein than those with chronic disease (*K*_D_ = 1.6 × 10^−10^ M vs. 3.0 × 10^−10^ M) but this was not statistically significant (E). (GT1a HCV infected patient *n* = 47, GT3a HCV infected patient *n* = 20). ELISA, enzyme‐linked immunosorbent assay; HCV, hepatitis C virus; SD, standard deviation.

The average anti‐E2 IgG antibodies in plasma of all patients with HCV of 97.6 µg/mL (range 0.41–1202 µg/mL was significantly higher than the 0.09 µg/mL measured in plasma of healthy controls (mean = 0.04 ± 0.007 µg/mL; *p* = 0.0001), but there was no significant difference in patients infected with GT1a virus (101.4 ± 30.1 µg/mL) than those infected with GT3a virus (88.9 ± 32.3 µg/mL; *p* = 0.2) (Figure 1A). Stratification of the whole cohort by clinical outcome showed that patients with chronic disease had 11.9 times higher anti‐E2 IgG than those who cleared the viruses with mean concentrations of 169.5 ± 39.8 versus 14.3 ± 3.5 µg/mL, respectively. Further substratification by infecting viral genotype and the clinical outcome showed that patients with chronic disease due to GT1a virus infection had an average of 17 times higher (188.5 ± 53.5 vs. 11.1 ± 3.8 µg/mL) and patients with chronic disease due to GT3a virus infection had 5.6 times higher (132.5 ± 50.6 vs. 23.5 ± 7.5 µg/mL) antibody concentrations than patients that cleared their corresponding viral genotypes, indicating disease chronicity was the main driver of high antibody responses (Figure 1B,C). Further analysis of the antibody subtypes and IgG subclasses showed that anti‐E2 IgG1 was the most abundant, and those with chronic disease had significantly more IgG1 than the clearers (*p* = 0.01; Figure 1D). Similar trends were also observed for IgM and most patients had detectable IgA, however, there was little to no detectable IgG2, IgG3, and IgG4 in both patient groups (Figure 1D). Measurement of the interaction of the plasma anti‐E2 antibodies with E2 protein using SPR showed that on average antibodies from patients that cleared the virus had approximately two times higher affinity to E2 protein than those with chronic disease with an average binding affinity (*K*_D_) of 1.6 × 10^−10^M ± 8.0 SEM compared to 3.0 × 10^−10^ M ± 9.2 SEM, although this was not statistically significance (*p* = 0.07) (Figure 1E). Detailed data of the association (ka or kon), dissociation (kd or koff) and equilibrium (*K*_D_) for each patient is shown in Table 2 (SPR sensorgrams available on request).

**Table 2:** Characterization of the interaction between plasma anti‐E2 antibodies and E2 protein by SPR.

Characteristics of interaction	Patients	Chronic (*n* = 24)	Clearer (*n* = 23)
Association constant (ka) (Ms^−1^)	Pt 1	2.74E+04	2.00E+05
	Pt 2	4.73E+04	2.05E+04
	Pt 3	8.36E+04	8.31E+04
	Pt 4	3.74E+04	5.96E+05
	Pt 5	7.38E+04	9.13E+04
	Pt 6	7.35E+05	2.22E+05
	Pt 7	7.26E+03	2.07E+05
	Pt 8	8.34E+04	4.08E+05
	Pt 9	7.26E+04	2.95E+05
	Pt 10	7.37E+05	8.31E+05
	Pt 11	8.83E+05	2.95E+05
	Pt 12	1.31E+05	5.64E+05
	Pt 13	2.52E+05	6.55E+05
	Pt 14	2.63E+05	3.80E+04
	Pt 15	9.37E+04	5.15E+05
	Pt 16	5.01E+04	4.72E+04
	Pt 17	2.53E+04	3.59E+05
	Pt 18	7.13E+04	4.25E+04
	Pt 19	8.72E+04	9.59E+05
	Pt 20	1.31E+04	3.80E+05
	Pt 21	3.53E+04	4.31E+05
	Pt 22	2.67E+05	7.19E+04
	Pt 23	1.42E+05	3.90E+05
	Pt 24	6.25E+04	
Dissociation constant (kd) (s^−1^)	Pt 1	3.26E−09	2.10E−04
	Pt 2	2.96E−07	6.23E−06
	Pt 3	3.66E−06	1.43E−05
	Pt 4	1.01E−09	2.19E−06
	Pt 5	1.95E−07	3.30E−07
	Pt 6	1.65E−04	1.10E−07
	Pt 7	6.89E−06	2.06E−05
	Pt 8	2.62E−06	2.28E−06
	Pt 9	3.71E−06	7.66E−07
	Pt 10	1.02E−03	8.29E−06
	Pt 11	1.31E−03	2.70E−06
	Pt 12	3.36E−07	7.89E−04
	Pt 13	4.61E−05	1.41E−07
	Pt 14	7.35E−05	1.41E−08
	Pt 15	8.31E−08	6.39E−06
	Pt 16	9.07E−07	2.02E−09
	Pt 17	1.58E−06	2.24E−07
	Pt 18	4.53E−05	5.39E−07
	Pt 19	2.74E−07	6.33E−04
	Pt 20	9.55E−06	2.37E−05
	Pt 21	9.01E−07	1.38E−06
	Pt 22	1.96E−06	3.27E−06
	Pt 23	4.95E−05	2.99E−06
	Pt 24	5.15E−05	
Binding affinity (*K*_D_) (M)	Pt 1	1.19E−13	1.05E−09
	Pt 2	6.26E−12	3.03E−10
	Pt 3	4.38E−11	1.72E−10
	Pt 4	2.71E−14	3.67E−12
	Pt 5	2.65E−12	3.61E−12
	Pt 6	2.24E−10	4.93E−13
	Pt 7	9.49E−10	9.95E−11
	Pt 8	3.14E−11	5.6E−12
	Pt 9	5.11E−11	2.6E−12
	Pt 10	1.38E−09	9.98E−12
	Pt 11	1.48E−09	9.13E−12
	Pt 12	2.56E−12	1.4E−09
	Pt 13	1.83E−10	2.15E−13
	Pt 14	2.8E−10	3.7E−13
	Pt 15	8.87E−13	1.24E−11
	Pt 16	1.81E−11	4.29E−14
	Pt 17	6.26E−11	6.24E−13
	Pt 18	6.36E−10	1.27E−11
	Pt 19	3.14E−12	6.6E−10
	Pt 20	7.27E−10	6.24E−11
	Pt 21	2.55E−11	3.21E−12
	Pt 22	7.32E−12	4.55E−11
	Pt 23	3.48E−10	7.68E−12
	Pt 24	8.24E−10	

Abbreviation: SPR, surface plasmon resonance.

### 2.3 Patients with HCV infection have ADCP, and those with chronic disease have significantly higher phagocytic and neutralization functions than clearers

**Figure 2:**
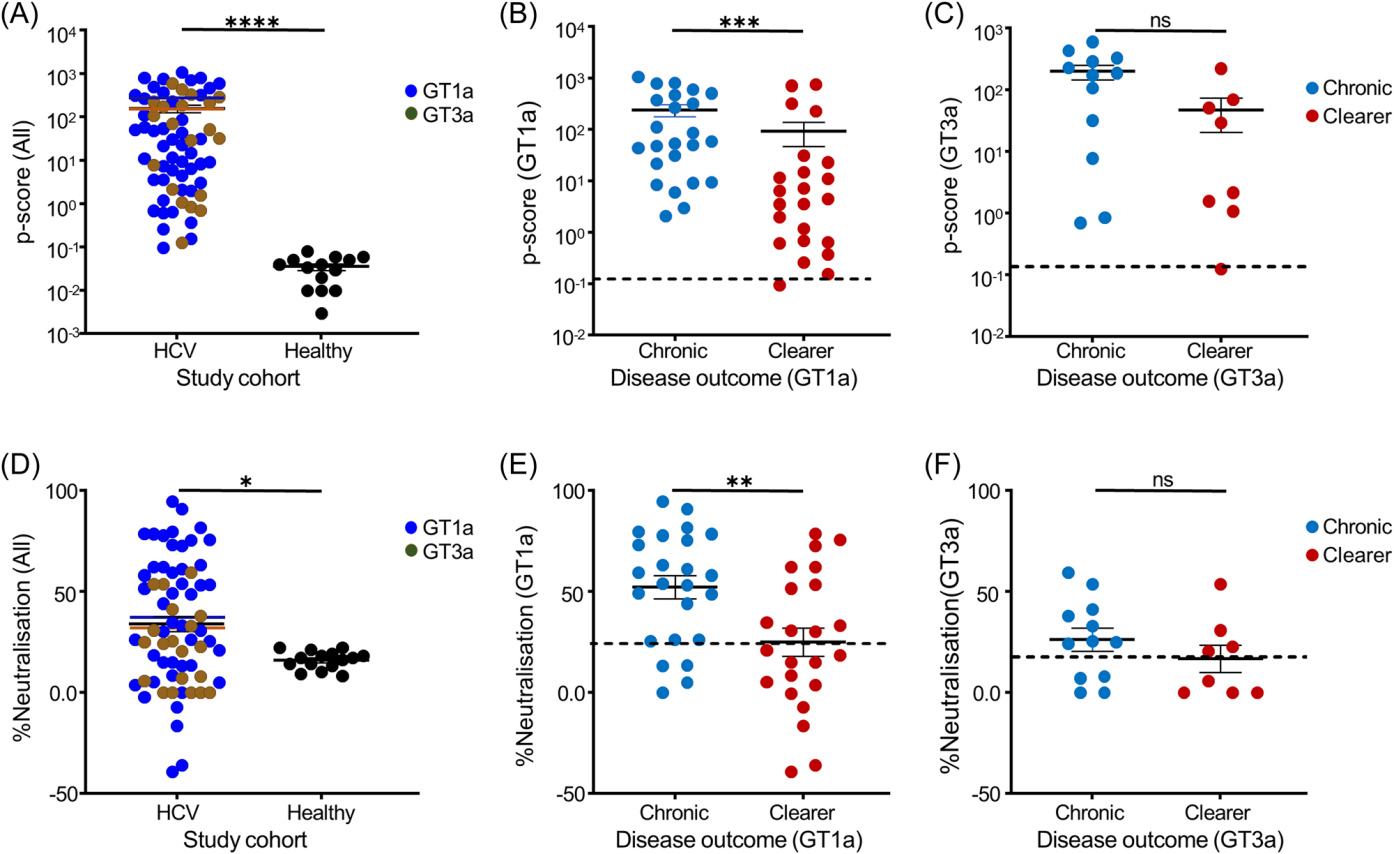
Antibody‐dependent cellular phagocytosis (ADCP) of HCV E2 protein‐coated patient plasma opsonised microbeads and neutralization of HCVpp. Most patients (97%) infected with HCV had measurable phagocytic functions with a mean p‐score of 158.8 ± 30.3 compared to healthy controls (mean p‐score = 0.03 ± 0.01); *p* = 0.0001) and those infected with GT1a virus showed higher p‐score (mean = 171.6 ± 36.7) compared with those infected with GT3a virus (mean p‐score = 138.0 ± 37.9) (A). Stratification by the clinical outcome and infecting viral genotype showed that patients with chronic disease due to GT1a virus infection showed significantly higher phagocytosis (mean p‐score = 239.6 ± 63.6) than those who cleared the virus with (mean p‐score = 92.5 ± 44.9) (*p* = 0.0001) (B). Similarly, patients with chronic disease due to GT3a virus infection had higher p‐score (mean p‐score = 198.7 ± 54.4) compared to those who cleared the virus (mean p‐score = 46.9 ± 26.4), although not statistically significant (*p* = 0.06) (dotted lines indicate an average value from 15 healthy controls +3 SD) (C). Plasma from 64% of patients had positive neutralization effects of GT1a or GT3a HCVpp as defined as percentage neutralization at least 2 SD above the mean value of the healthy controls and those infected with the GT1a virus had better neutralization function (mean = 39.7 ± 4.8%) than those infected with the GT3a virus (mean = 22.4 ± 4.3%) (*p* = 0.02) (D). Stratification by the clinical outcome and infecting viral genotype showing chronically infected patients with the GT1a virus had significantly better neutralization function (mean = 52.1 ± 5.6%) than those who cleared the virus (mean = 24.8 ± 6.9%) (*p* = 0.008) (E). Similarly, chronically infected patients with the GT3a virus had better neutralization function (mean = 26.2 ± 5.7%) than those who cleared the virus (mean = 16.7 ± 6.7%) but this was not statistically significant (*p* = 0.2) (dotted lines indicate an average value from 15 healthy controls+3 SD) (F). A negative neutralization percentage refers to an enhancement of infection. (GT1a HCV infected patient *n* = 47, GT3a HCV infected patient *n* = 20). HCV, hepatitis C virus; SD, standard deviation.

Phagocytosis assay of the plasma‐opsonised microbeads showed that 97% of patients had positive phagocytic functions defined as at least 3 standard deviations above the mean *p*‐score of 0.03 ± 0.01 in healthy controls. The average p‐score in the whole patient cohort was 158.8 ± 30.3 with those infected with the GT1a virus showing a measurably higher mean p‐score of 171.6 ± 36.7 compared to those infected with GT3a virus (mean p‐score = 138.0 ± 37.9) (Figure 2A). Stratification of the whole cohort by clinical outcome showed those with chronic disease had significantly higher phagocytic function with a mean p‐score of 226.0 ± 45.8 than those who cleared the viruses (mean p‐score = 80.7 ± 34.0) (*p* = 0.0001). Further, stratification by infecting viral genotype and the clinical outcome showed that patients with chronic disease due to GT1a virus infection showed significantly higher phagocytic functions than those who cleared the virus with average p‐scores of 239.6 ± 63.6 and 92.5 ± 44.9, respectively (*p* = 0.0001; Figure 2B). Similarly, patients with chronic disease due to GT3a virus infection had a higher average p‐score of 198.7 ± 54.4 compared to 46.9 ± 26.4 in those who cleared the virus, although this was not statistically significant (*p* = 0.06; Figure 2C).

Neutralization assay with the GT1a or GT3a HCV pseudoviruses (HCVpp) showed plasma from 43/67 (64%) of patients had positive neutralization activity (Figure 2D). The mean positive neutralization function in patients infected with the GT1a virus was significantly higher than those infected with the GT3a virus (39.7 ± 4.8% vs. 22.4 ± 4.3%) (*p* = 0.02). Interestingly, in 5/67 patients (all GT1a infected), enhancement of HCVpp infection was observed denoted by the negative neutralization values. Stratification by clinical outcome showed patients with chronic disease had significantly better neutralization activity than clearers with mean values of 43.5 ± 4.6% and 22.8 ± 5.4%) respectively (*p* = 0.007). Further sub‐stratification based on the clinical outcome and infecting viral genotype showed chronically infected patients with the GT1a had significantly better neutralization function (mean = 52.1 ± 5.6%) than those who cleared the virus (mean = 24.8 ± 6.9%) (*p* = 0.008; Figure 2E). Similarly, chronically infected patients with GT3a had better neutralization function (mean = 26.2 ± 5.7%) than those who cleared the virus (mean = 16.7 ± 6.7%) (*p* = 0.2; Figure 2F). These results indicate that patients with chronic disease had better ADCP and neutralization functions regardless of the genotype of the infecting HCV, likely due to the higher levels of anti‐E2 antibodies observed in their circulation (Figure 1).

### 2.4 Older patients have higher anti‐E2 antibody levels and better ADCP and neutralization function than younger patients

**Figure 3:**
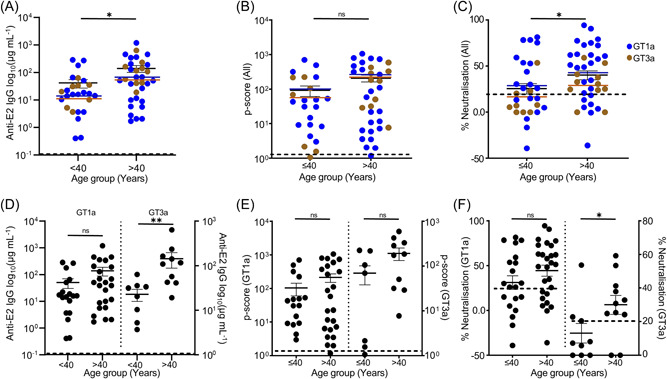
Age‐dependent differences in anti‐E2 antibody levels, ADCP, and neutralization functions. Plasma anti‐E2 antibody concentration in the over 40‐year‐old patients was significantly higher (mean = 140.4 ± 38.1 µg/mL) than those under 40 years of age (mean = 41.7 ± 14.0 µg/mL) (*p* = 0.03) (A) Analysis of the phagocytic and neutralization functions showing older patients had higher p‐scores (mean = 208.9 ± 46.6 vs. 93.1 ± 31.2; *p* = ns) (B) and better neutralization function (mean = 40.3 ± 4.8% vs. 25.6 ± 5.8%; *p* = ns) (C). These age‐dependent differences in anti‐E2 antibody concentration, phagocytosis, or neutralization were maintained regardless of the infecting viral genotype with older patients infected with GT1a having 2.7 times more antibodies (mean = 139.4 ± 49.1 vs. 50.2 ± 20 µg/mL; *p* = ns) (D left), 2.1 times higher p‐score (215.1 ± 61.2 vs. 103.5 ± 43.2; *p* = ns) (E left) and 1.4 times better neutralization function (mean = 44.4 ± 6.2% vs. 31.2 ± 7.7%; *p* = ns) (F left). Similarly, older patients infected with GT3a virus showed 15.9 times more antibodies (143 ± 54.2 vs. 22.8 ± 6.9 µg/mL; *p* = 0.009) (D right), 2.8 times higher p‐score (193.7 ± 59.7 vs. 69.9 ± 31.2; *p* = ns) (E right) and 2.3 times better neutralization (30 ± 5.6% vs. 13.1 ± 5.8%; *p* = 0.04) (F right). The dotted lines indicate the average value from 15 healthy controls. Negative neutralization values indicate enhancement of infection. (GT1a HCV infected patient *n* = 47, GT3a HCV infected patient *n* = 20). ADCP, Antibody‐dependent cellular phagocytosis; HCV, hepatitis C virus.

Patients who are over 40‐year‐old had significantly more plasma anti‐E2 antibodies (mean = 140.4 ± 38.1 µg/mL) than those under 40 years of age (mean = 41.7 ± 14.0 µg/mL) (*p* = 0.03; Figure 3A). Phagocytic and neutralization functions were also higher in the older than the younger patients with mean p‐scores of 208.9 ± 46.6 versus 93.1 ± 31.2 and mean neutralization function of 40.3 ± 4.8% versus 25.6 ± 5.8% respectively (Figure 3B,C). These significant age‐dependent differences in anti‐E2 antibody concentration, and neutralization were maintained for GT3a, where the older patients showed 15.9 times more antibodies (mean = 143 ± 54.2 vs. 22.8 ± 6.9 µg/mL), and 2.3 times better neutralization (30 ± 5.6% vs. 13.1 ± 5.8%) (Figure 3D,F). Although not statistically significant, older patients infected with GT1a had also 2.7 times more antibodies (mean = 139.4 ± 49.1 vs. 50.2 ± 20 µg/mL), 2.1 times higher phagocytic score (mean = 215.1 ± 61.2 vs. 103.5 ± 43.2) and 1.4 times better neutralization function (44.4 ± 6.2% vs. 31.2 ± 7.7%) (Figure 3D–F). Interestingly, there was no significant difference in the concentration of plasma anti‐E2 antibodies, phagocytosis or neutralization functions between males and females regardless of the viral genotype causing the infection (Supporting Information S1: Figure 1).

### 2.5 Plasma anti‐E2 antibody levels significantly correlated to ADCP and neutralization function

**Figure 4:**
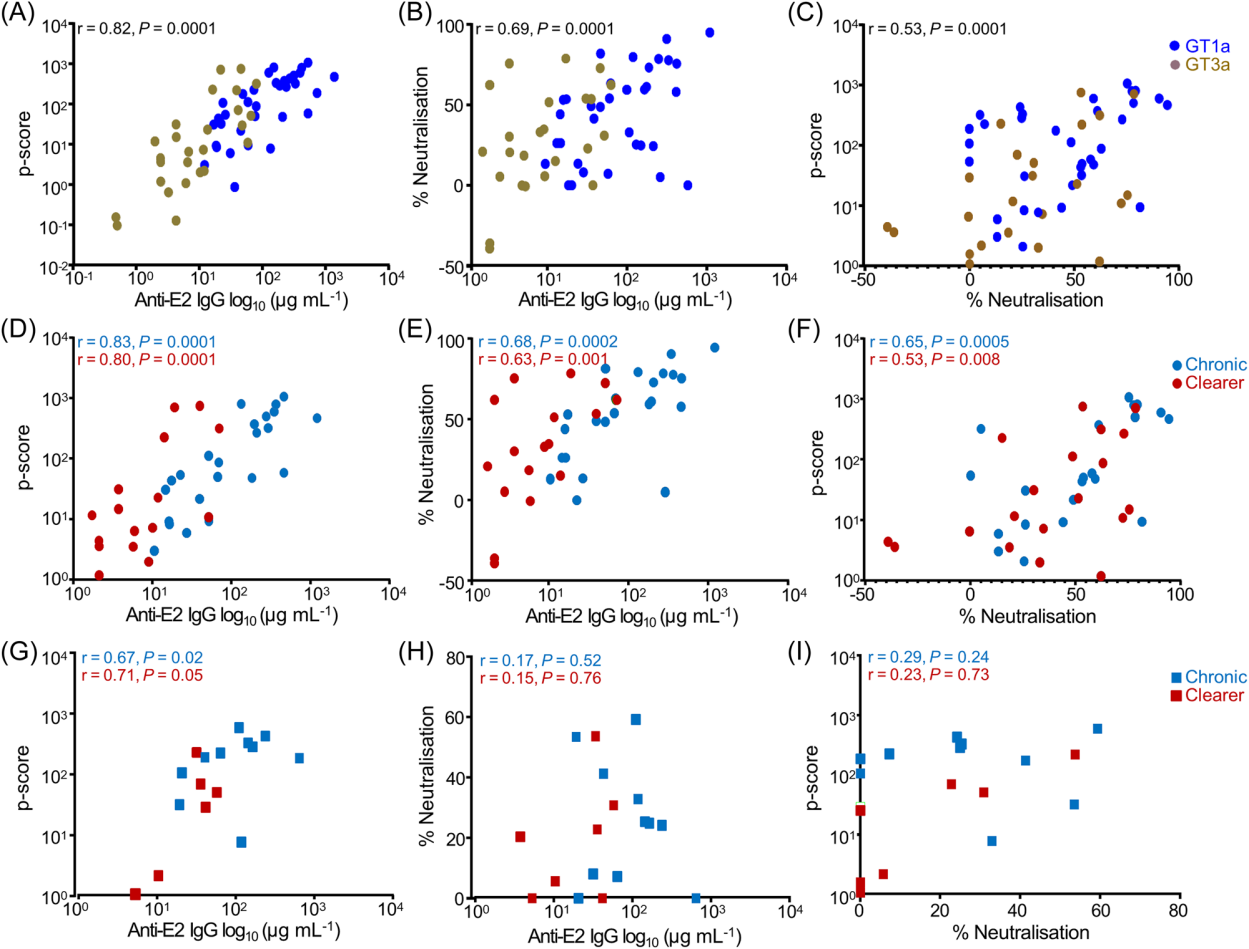
Correlation of patient plasma anti‐E2 antibody levels with ADCP and neutralization functions. Spearman correlation of all patient cohorts showing a significant positive correlation between ADCP and anti‐E2 antibody levels (*r* = 0.82, *p* = 0.0001) (A), between neutralization and anti‐E2 antibody levels (*r* = 0.69, *p* = 0.0001) (B) and between ADCP and neutralization function (*r* = 0.53, *p* = 0.0001) (C). Spearman correlation studies of patients infected with GT1a virus show a significant positive correlation between ADCP and anti‐E2 antibody levels (D), between neutralization and anti‐E2 antibody levels (E) and between ADCP and neutralization (F) in chronically infected patients with *r* = 0.83, *p* = 0.0001; *r* = 0.68, *p* = 0.0002 and *r* = 0.65, *p* = 0.0005 respectively (shown in blue) and those who cleared the virus with *r* = 0.80, *p* = 0.001; *r* = 0.63, *p* = 0.001; *r* = 0.53, *p* = 0.008 respectively (shown in red). In patients infected with GT3a virus there was significant positive correlation between ADCP and anti‐E2 antibody levels in both chronically infected patients (*r* = 0.67, *p* = 0.02) and those who cleared the virus (*r* = 0.71, *p* = 0.05) (G) but there were no significant correlations between neutralization and anti‐E2 antibody levels (H) or between ADCP and neutralization functions (I). GT1a HCV infected patient *n* = 47, GT3a HCV infected patient *n* = 20). ADCP, Antibody‐dependent cellular phagocytosis; HCV, hepatitis C virus.

Spearman correlation studies of the whole patient cohort showed that anti‐E2 level in the plasma positively correlated with ADCP (*r* = 0.82, *p* = 0.0001) and neutralization function (*r* = 0.69, *p* = 0.0001) (Figure 4A,B). There was also a significant positive correlation between ADCP and neutralization function (*r* = 0.53, *p* = 0.0001) (Figure 4C), indicating these two functions are dependent on the antibody titer. The significant positive correlation between the antibody levels and ADCP was retained in patients infected with the GT1a virus whether they had chronic disease (*r* = 0.83, *p* = 0.0001) or had cleared the virus (*r* = 0.80, *p* = 0.001) (Figure 4D). The significant positive correlation between the antibody levels and neutralization was also retained in those who developed the chronic disease (*r* = 0.68, *p* = 0.0002) and those who cleared the virus (*r* = 0.63, *p* = 0.001) (Figure 4E). Similar results were observed when analysing the relationship between ADCP and neutralization in both patient groups (Figure 4F). In patients infected with the GT3a virus, a significant positive correlation was observed between antibody levels and ADCP in both patients with chronic disease and those who cleared the virus (Figure 4G). However, the correlations between antibody levels and neutralization (Figure 4H) as well as between ADCP and neutralization (Figure 4I) were not statistically significant.

### 2.6 Affinity of the plasma anti‐E2 antibodies positively correlated with ADCP in patients who cleared GT1a virus but not in those with chronic disease

**Figure 5:**
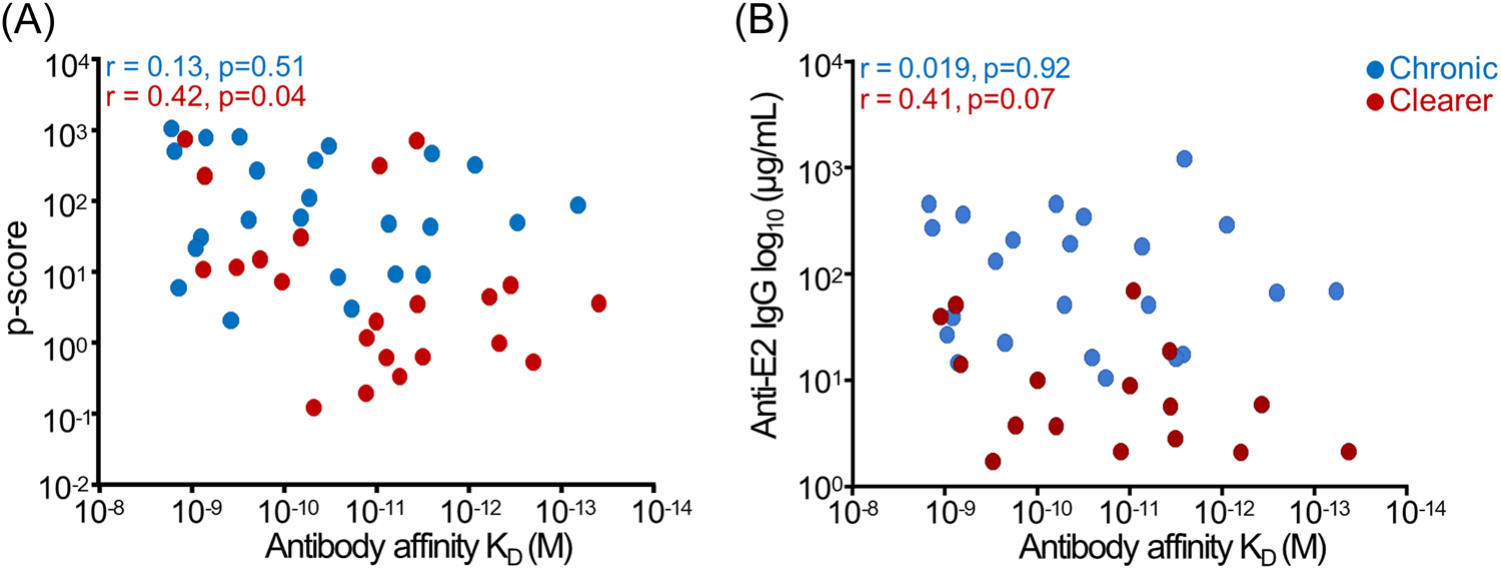
Correlation between ADCP and affinity of the anti‐E2 antibodies in plasma of patients infected with GT1a HCV. There was significant positive correlation between ADCP and the affinity of the plasma antibodies to HCV E2 protein and ADCP in patients who cleared the virus but not those with chronic disease with *r* = 0.42 and a *p*‐value of 0.04 compared with the nonsignificant relationship in those with chronic disease *(r* = 0.13, *p* = 0.5) (A). By contrast, there was no correlation between affinity and antibody titers in both patients who cleared the virus and those with chronic disease (B), suggesting the significant relationship between ADCP and affinity in clearers was independent of the antibody titer. (Patients with chronic GT1a HCV infection *n* = 25; patients that clear the virus clearers *n* = 24). ADCP, Antibody‐dependent cellular phagocytosis; HCV, hepatitis C virus.

There was significant positive correlation between the affinity of the plasma antibodies to HCV E2 protein and ADCP in patients who cleared the virus but not in those with chronic disease. The significant positive correlation between affinity and ADCP in those who cleared the virus was *r* = 0.42, *p* = 0.04 compared to the nonsignificant correlation in those with chronic disease (*r* = 0.13, *p* = 0.51) (Figure 5A). Interestingly, there was no significant correlation between antibody titers and affinity in both patients with chronic disease and those who cleared the virus, suggesting differences in affinity were independent of antibody concentrations in the plasma (Figure 5B). This result suggests that ADCP was maintained in clearers due to the affinity (quality) of their anti‐E2 antibodies despite having lower antibody titers.

### 2.7 Epitope mapping and correlation matrices of the associative relationship of ADCP to multiple independent variables

**Figure 6:**
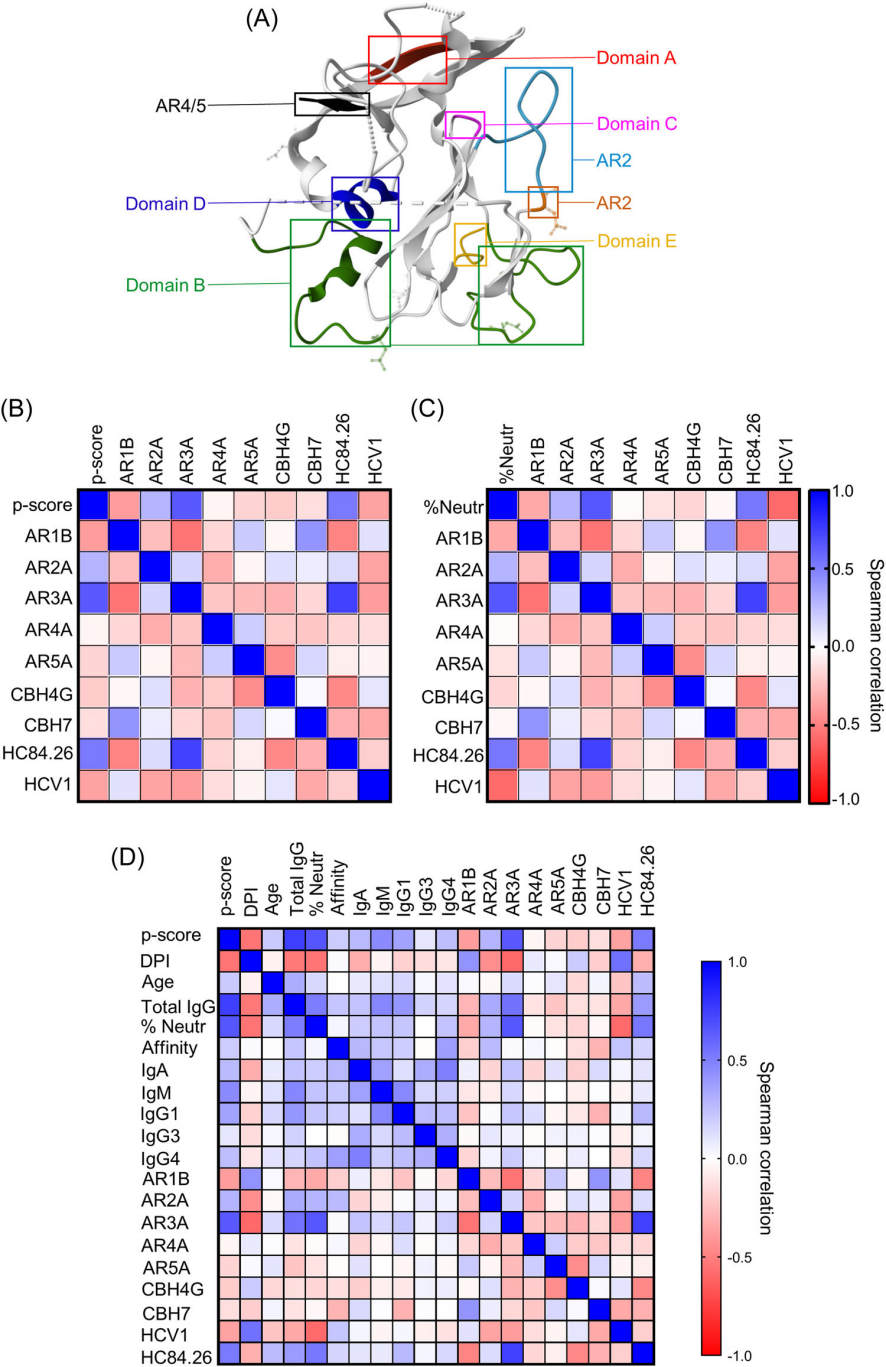
Epitope mapping and parameter matrices of the associative relationship of ADCP to multiple independent variables. Schematic illustration of the HCV envelope protein E2 with the different Domains (Dom) and/or antigenic regions (AR) targeted by specific monoclonal antibodies used for epitope mapping of plasma anti‐E2 antibodies produced by patients: Domain A (red; mAb CBH4G), Domain B (green; mAb AR3A), Domain C (pink; mAb CBH7), Domain D (blue; mAb HC84.26), Domain E (yellow; mAb HCV1), region AR1 (cyan; mAb AR1B), region AR2 (orange; mAb AR2A), region AR4/5 (black; mAb AR4/5 A)^30^ (A). A heatmap of a parameter correlation matrix between ADCP and the different E2 epitopes targeted by antibodies in patient plasma shows that antibodies targeting Domain B (AR3A) and Domain D (HC84.26) had a significant positive relationship with high phagocytosis with *r* scores of 0.64 (*p* = 0.0001) and 0.51 (*p* = 0.0003), respectively as contrasted to the significant negative relationships observed with antibodies targeting Domain E (HCV1) and Domain C (CBH‐7) with *r* scores of −0.38 (*p* = 0.008) and −0.36 (*p* = 0.0003) respectively (B). A heatmap of a parameter correlation matrix between neutralization function and the different E2 epitopes targeted by antibodies in patient plasma also showed that antibodies targeting Domains B and D also had a significant positive association to neutralization function with *r* scores of 0.65 (*p* = 0.0001) and 0.53 (*p* = 0.0004) respectively while those targeting Domains E (*r* = −0.33; *p* = 0.03) and Domain C (*r* = −0.58; *p* = 0.0004) had significant negative associations, suggesting functional overlap between ADCP and neutralization (C). Further analysis of the associative relationship inputting all the relevant continuous variables indicated that levels of anti‐E2 IgG (*r* = 0.75; *p* = 0.0001), neutralization function (*r* = 0.66; *p* = 0.0001), antibodies in patient plasma targeting Domains B (*r* = 0.64; *p* < 0.0001), Domain D (*r* = 0.51; *p* = 0.0003), anti‐E2 IgG1 (*r* = 0.36; *p* = 0.01), anti‐E2 IgM (*r* = 0.45; *p* = 0.001) and anti‐E2 IgA (*r* = 0.27; *p* = 0.06) showed a positive association with the phagocytic scores. By contrast, DPI (*r* = −0.54; *p* = 0.0005), antibodies targeting antigenic region 1 (AR1) (*r* = −0.38; *p* = 0.001) and antibodies targeting Domain E (*r* = −0.36; *p* = 0.01) showing negative associations (D). (Patients with chronic GT1a HCV infection *n* = 25; patients that clear the virus clearers *n* = 24).

The HCV envelope protein E1E2 with the different domains (Dom) and/or antigenic regions (AR) targeted by specific monoclonal antibodies used for epitope mapping of plasma anti‐E2 antibodies produced by patients is shown in Figure 6A for reference.^30^ A statistical correlation matrix showed that antibodies in patient plasma targeting E2 Domain B (AR3A) and Domain D (HC84.26) had a significant positive relationship with high phagocytosis with *r* scores of 0.64 (*p* = 0.0001) and 0.51 (*p* = 0.0003), respectively, as contrasted to the significant negative relationships observed between high phagocytosis and antibodies targeting E2 Domain E (HCV1) and Domain C (CBH‐7) with *r* scores of −0.38 (*p* = 0.008) and −0.36 (*p* = 0.0003), respectively, (Figure 6B). Interestingly, antibodies targeting Domains B and D had also a significant positive association to neutralization function with *r* scores of 0.65 (*p* = 0.0001) and 0.53 (*p* = 0.0004), respectively, while those targeting Domains E (*r* = −0.33; *p* = 0.03) and Domain C (*r* = −0.58; *p* = 0.0004) had significant negative associations, suggesting functional overlap between ADCP and neutralization (Figure 6C). These results suggest that patients produced antibodies with overlapping/shared phagocytic and neutralization functions. Further analysis of the associative relationship by inputting all the relevant continuous variables indicated that levels of anti‐E2 IgG (*r* = 0.75; *p* = 0.0001), neutralization function (*r* = 0.66; *p* = 0.0001), anti‐E2 IgG1 (*r* = 0.36; *p* = 0.01), anti‐E2 IgM (*r* = 0.45; *p* = 0.001) and anti‐E2 IgA (*r* = 0.27; *p* = 0.06) showed a positive association with the phagocytic scores. By contrast, antibodies targeting E2 antigenic region 1 (AR1B) (*r* = −0.38; *p* = 0.001) or Domain E (*r* = −0.36; *p* = 0.01) showed negative associations (Figure 6D).

## 3 DISCUSSION

This study demonstrated that all patients in this cohort had detectable anti‐E2 antibodies in their plasma, predominantly the IgG1 subclass, and those with chronic disease showed significantly higher antibody concentrations than those that cleared the virus. These results are consistent with previous studies in HCV in which high antibody titers are associated with chronic disease^31^, ^32^ and IgG1 is one of the most dominant subclasses.^33^, ^34^ This is consistent with previous reports on patients infected with HIV in which patients with chronic disease displayed high IgG1 titers.^35^

We found that patients who cleared the virus had antibodies with higher affinities compared to those with chronic HCV disease, albeit not reaching statistical significance. Moreover, there was a significant correlation between affinity and ADCP in those who cleared the virus but not in patients with chronic disease, suggesting that the former may have better quality antibodies. This trend is different to that reported previously where patients who cleared infection were found to have antibodies with lower somatic hypermutation (and lower affinity) compared to chronics.^36^, ^37^ However, we need to consider the fact, that these studies utilized the patient memory B cell‐derived monoclonal antibodies, not plasma antibodies. A potential mechanism underlying the higher affinity antibodies in the patients that cleared the virus may include superior CD4 T follicular help. Studies in primary HCV infection have reported increased circulating T follicular help coincides with early HCV E2 neutralizing Abs in HCV clearers.^38^ Lower affinity in patients with chronic disease may also in part be explained by more mutations in the E2 protein further than the consensus sequence of the recombinant E2 proteins used in this study. However, the study team's unpublished longitudinal study showed that both patients with chronic disease and those that cleared the virus maintained high affinity binding to the same E2 protein for over 400 days postinfection, suggesting mutations had a comparable impact to the core immunogenic domains of the E2 proteins in both groups.

Here we show for the first time that patients with HCV infection can mount ADCP regardless of the genotype of infecting virus, disease outcome, or sex. Further characterization of the determinants of the magnitude of ADCP responses indicated that older patients with chronic HCV infection with high antibody titers showed significantly higher phagocytic scores when compared to those patients who cleared the virus.

In this study we confirmed previous reports showing the positive correlation between neutralization and high antibody titers in patients infected with HCV^39^, ^40^ as well as in HIV,^18^, ^41^ however, this is the first to demonstrate a significant positive correlation between ADCP and high anti‐E2 antibody titers, particularly in patients with chronic disease. Importantly, we also found a significant positive correlation between ADCP and affinity in only patients who clear the virus but not in those with chronic disease, indicating that while high antibody titer was the main driver of ADCP in patients with chronic disease, better quality antibodies may have contributed to ADCP in those who cleared the virus.

It is noteworthy that although ADCP assay using antigen‐coated microbeads is a highly feasible and widely accepted surrogate in diverse infections that require access to high‐level Physical Containment laboratories (PC3) including Mycobacteria,^42^ HIV,^43^ SARS‐COV‐2,^44^ and Influenza,^45^ absence of live organisms in the assay system is a major limitation. Similarly, the use of recombinant HCV E2 for ELISA^46^ and HCV E1E2 pseudovirus for neutralization,^47^ are also well‐established surrogates of anti‐HCV antibody characterization. Particularly in HCV, multiple genotypes and high levels of heterogeneity due to rapid mutations leading to millions of quasispecies within a single individual as well as technical difficulties of culturing primary viral isolates are further limiting factors.

We found that anti‐E2 antibodies in the plasma of patients that target the antigenic Domains B and D of the viral envelope strongly correlated with high ADCP and neutralization function, suggesting antibodies generated in responses to these antigenic domains may possess superior effector functions compared to other regions on the viral envelope. Interestingly, Domains B and D are the primary response targets and are known to represent most antibodies present in sera of patients infected with HCV^13^, ^33^ (Supporting Information S2: Figure 2), thus overrepresentation of these antibodies in the circulation may, in part, explain their strong positive association to ADCP and neutralization functions. A vaccine(s) designed to provoke antibody responses against these two antigenic Domains may therefore induce high titer antibodies that have dual ADCP and broad neutralization functions, thereby providing better protection.

The potential immunological role of ADCP involves the clearance of pathogens and pathogen‐infected cells, activation of adaptive immune responses by facilitating antigen presentation, and induction of inflammatory mediators.^45^ Mechanistically, ADCP mostly leads to destruction of the phagocytosed pathogens or infected cells via phagolysosome‐mediated degradation, and clinically there is convincing evidence for the protective role of ADCP in other RNA viruses such as SARS‐CoV‐2 ^48^ and HIV.^49^ However, some pathogens such as Dengue, Yellow fever virus, and Zika virus may evade degradation leading to antibody‐dependent enhancement (ADE) of infection with potentially deleterious clinical effects.^50^, ^51^, ^52^, ^53^, ^54^ In HCV, it is not clear yet if ADCP is beneficial or deleterious, however, the finding showing its strong positive correlation with neutralization function, which is known to confer protection against the disease may indicate ADCP also plays a beneficial role. Moreover, we found that patients with anti‐E2 antibodies targeting Domain B and D that are known to be associated with effective neutralization^55^, ^56^, ^57^, ^58^, ^59^ are also the ones that had antibodies strongly associated with high ADCP, further supporting the notion that there may be overlapping protective functions via dually acting antibodies. This is consistent with reports on HIV in which overlapping ADCP and neutralizing functions were observed,^24^ and antibodies that maintain broad neutralization function against several HIV variants also possessed potent Fc receptor‐dependent effector functions including ADCP.^14^, ^17^, ^24^, ^25^ However, future studies aimed at defining the significance of ADCP in HCV, namely its role in viral clearance and clinical outcomes using live viruses, is warranted.

Taken together, this study demonstrated ADCP and neutralization in patients with chronic HCV infection and in those that cleared the virus that was likely mediated by dual‐function antibodies that target shared epitopes. Patients that cleared the virus displayed lower titer but higher affinity antibodies that strongly correlated with ADCP, suggesting that ADCP was maintained in clearers due to the quality (affinity), while it was mostly driven by antibody titer in patients with chronic disease.

## 4 METHODS

### 4.1 Study cohort and sample size calculation

The Hepatitis C Incidence and Transmission Study (HITS) cohort enrolled 621 high‐risk (injecting drug users) HCV seronegative participants in prisons (HITS‐p) and the general community (HITS‐c) from 34 sites across New South Wales, Australia, between 2005 and 2016 and prospective blood samples were collected every 3–6 months with HCV RNA and seroconversion monitored.^60^ Upon infection, the HCV‐positive individuals were sampled every 24 weeks. In this study, frozen plasma and sera from 47 individuals who were infected with genotype 1a (GT1a) and 20 individuals infected with genotype 3a (GT3a) HCV from the HITS‐p cohort were collected to assess anti‐HCV envelope 2 (E2) antibody subclasses and to measure antibody titers, ADCP, neutralization function, and affinity. Forty‐one GT1a and 18 GT3a samples were collected between 50 and 250 days postinfection (DPI), whereas samples of six individuals with HCV GT1a infection and two individuals with HCV GT3a infection were collected between 16.5 and 50 DPI and 12–50 DPI, respectively. The date each individual patient is confirmed to be infected is determined by the initial day on which they test positive for the anti‐HCV IgM antibody through either anti‐HCV IgM antibody test and/or HCV RNA via real‐time PCR. Subsequent days are counted from this initial date and referred to as “DPI.” Plasma from 15 healthy blood donors from the Australian Red Cross was used as control.

The primary study outcome was to assess differences in ADCP levels between patients and healthy controls, thus this variable was selected for sample size estimation. Based on a recent study that found an average 12. Fivefold increase in ADCP in patients with HCV than in the healthy controls,^61^ this variable set at a conservative fivefold difference, and 98% of patients with HCV expected to show 5fold higher ADCP, a sample size of 23 GT1a, 23 GT3a HCV‐infected patients and 15 healthy controls have 95% power at 5% two‐sided alpha (*α*) to detect statistically significant differences in mean values.^62^ The participants were genotyped for HCV as described previously.^50^

The study was approved by the New South Wales Justice Health Human Research Ethics Committees (G304/11), New South Wales Department of Corrective Services (11/103694), and the University of New South Wales (HC11579 and HC13237). Written informed consent was obtained from all participants.

### 4.2 Cells lines

The human embryonic kidney epithelial cell line 293T with stably knocked out CD81 (the main cell surface receptor for HCV entry), kindly donated by Joe Grove, University College London, UK, was used to produce GT1a and GT3a HCVpp. The lack of CD81 on these cells prevents attachment of HCVpp to their cell surface, thereby allowing accumulation of the HCVpp to the culture supernatant leading to increased yield. FreeStyle^TM^ 293 F cells (Thermo Fisher) were used to produce recombinant GT1a and GT3a HCV envelope proteins. The human hepatocyte cell line HuH‐7.5 with high CD81 expression obtained from Charles M. Rice, Rockefeller University, was infected with the GT1a or GT3a HCVpp and used to determine neutralizing activities of antibodies in the serum of patients infected with the corresponding viral genotype. The monocytic cell line THP‐1 (ATCC 202 TIB) was used as effector cells for Fc‐receptor mediated‐ADCP of patient plasma opsonized microbeads by flow cytometry as described.^28^

### 4.3 Culture of cell lines

The CD81 knockout 293T, 293F, and HuH‐7.5 cell lines were cultured at 2 × 10^4^ cells cm^−2^ in a 37°C and 5% CO_2_ in high glucose Dulbecco's Modified Eagle Medium (Gibco) supplemented with 10% v/v heat‐inactivated fetal bovine serum (FBS) (Gibco). 293T and HuH7.5 cells were passaged every 72 h upon reaching 80% confluency^63^ after detachment using TrypLE Express (Gibco), washing twice with phosphate‐buffered saline (PBS) and resuspension in fresh complete media, while the 293F that grew in suspension did not require the trypsinisation step. The CD81 knockout 293T cells and 293F cells were transiently transfected within 8–12 passages for the HCVpp and recombinant envelope protein production, respectively. The HuH7.5 cells were infected with HCVpp between 8 and 12 passages and used for the neutralization assays. For quality control, the expression of CD81 on the surface of the HuH7.5 and the CD81 knockout 293T cells was regularly assessed by immunofluorescence using an anti‐CD81 monoclonal antibody (mAb) (Santa Cruz Biotechnology).

THP‐1 cells were cultured at 5 × 10^4^ cells/mL at 37°C and 5% CO_2_ in RPMI 1640 supplemented with 2‐mM l‐glutamine (Gibco), 10% FBS, 0.05 mM β‐mercaptoethanol, 10 mM HEPES and 100 U/mL penicillin–streptomycin (Thermo Fisher). Expression of surface Fc‐receptors was assessed by flow cytometry using mAbs against Fcγ receptor RI (CD64)‐FITC, FcγRIII (CD16)‐PE, CD14‐PerCP (Becton Dickinson), and FcγRII (CD32)‐ACP (Life Technologies) and isotype and fluorochrome matched negative control mAbs (Becton Dickinson) and used for ADCP during passages 5–10.^61^

### 4.4 Production of recombinant HCV genotype 1a (GT1a) and GT3a envelope proteins

HCV exhibits high‐level heterogeneity within the same genotype and continuously mutates leading to millions of quasispecies within individuals, thereby creating logistical and technical challenges that require generation of hundreds of thousands of unique recombinant E2 proteins. This challenge was mitigated by producing a recombinant envelope 2 (E2) protein based on the consensus sequences from the H77 representing all the patients infected with GT1a and E2‐protein based on the consensus sequences from the UKN3A13.6 representing all the patients infected with GT3a virus were produced as described.^61^ In brief, the HCV E2 (amino acid residues 384–661) from GT1a or GT3a were cloned into a pcDNA3.1 mammalian expression vector with an N‐terminal secretion signal peptide and a C‐terminal Avi‐tag as well as a 6×histidine tag.^64^ Recombinant E2 proteins were then produced by transient transfection of 293 F cells followed by affinity purification and site‐specific biotinylation using the BirA biotin‐protein ligase kit (Avidity).^65^ Importantly, we confirmed that antibodies in the plasma obtained from all the patients in this study recognized these E2 proteins (antigens), and we selected patients with comparably high anti‐E2 antibody titers to undertake the Ab affinity and ADCP studies.

### 4.5 Quantification and isotyping of anti‐HCV E2 antibodies in the plasma of patients with HCV infection

Total anti‐HCV E2 IgG, IgG subclasses, IgA, and IgM in the plasma of the patients infected with HCV were quantified by a direct enzyme‐linked immunosorbent assay (ELISA).^33^ Briefly, to quantify total IgG, nunc immuno‐microtitre plates were coated with 500 ng of recombinant E2 per well for 2 h at room temperature, washed with 20 mM Tris‐HCl, pH 7.5, 150 mM NaCl, 0.1% Tween 20 (TBS‐T) and blocked overnight with 5% skim milk in TBS‐T at 4°C. Plates were washed with TBS‐T followed by dispensing of 50 µL per well of patient or control plasma diluted in 5% skim milk and incubation for 2 h at room temperature and two washes with the TBS‐T after that. Plate‐bound total anti‐E2 IgG antibodies were then detected with horse radish peroxidase (HRP)‐conjugated goat anti‐human polyclonal antibody and the IgG subclasses were detected using subclass specific‐HRP‐conjugated anti‐human antibodies (Jackson Immunoresearch) for 1 h at room temperature. To detect anti‐E2 IgA and IgM antibodies, HRP‐conjugated anti‐human IgA α‐chain‐specific and anti‐human IgM μ‐chain‐specific were used (Sigma‐Aldrich). These were followed by a colourimetric reaction to a 3,3′,5,5′‐tetramethylbenzidine horse radish peroxidase substrate for 10 min at room temperature, the addition of 1 M HCl stop buffer and measurement of optical density at 450 nm using BMG Labtech CLARIOstar microplate reader.

### 4.6 Coating of microbeads with GT1a or GT3a E2 proteins and opsonization with plasma anti‐E2 antibodies

Streptavidin‐tagged, Alexa 488 green florescent dye‐conjugated 0.4 µm Sphereotech polyester microbeads were surface coated with recombinant E2 proteins from GT1a or GT3a HCV and opsonized with plasma obtained from respective HCV genotype infected patients.^61^ Briefly, microbeads at a concentration of 1.5 × 10^9^ beads/mL were incubated with 50 μg/mL of biotinylated recombinant E2 proteins for 16 h at 4°C and excess proteins removed by washing the beads with LPS minimized cold PBS, pH 7.4. Aliquots (50 μL) of the E2‐coated beads were then incubated for 2 h at 37°C with 10 μL of plasma obtained from patients with HCV containing at least 0.1 μg/mL of anti‐E2 IgG or control plasma obtained from HCV negative healthy donors and resuspended in PBS. These opsonised microbeads were then stored in the dark at 4°C until used for ADCP assay.

### 4.7 Phagocytosis of GT1a or GT3a E2 protein‐coated‐ anti‐E2 antibody opsonized microbeads

**Figure 7:**
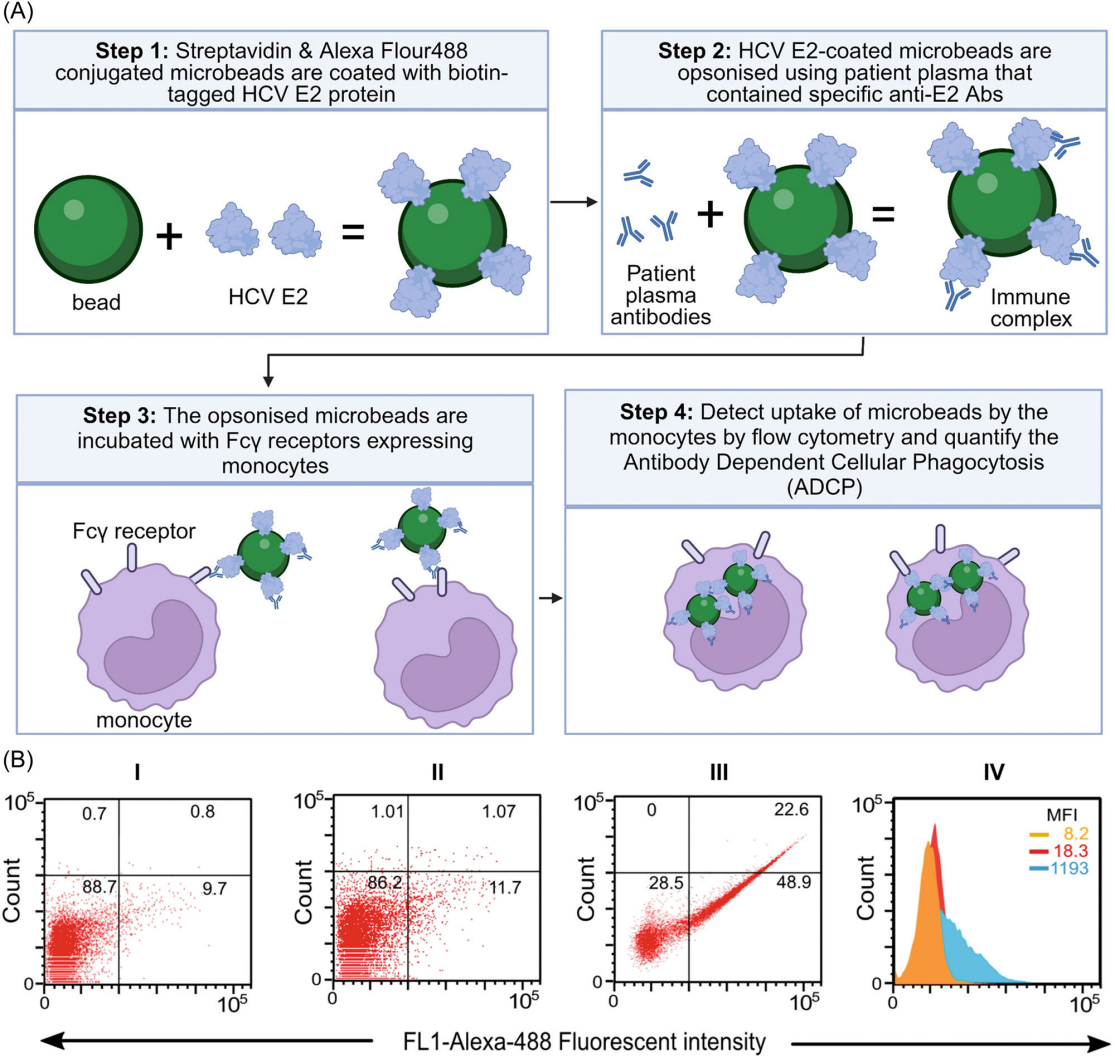
Antibody dependent cellular phagocytosis assay. Schematics showing step‐by‐step illustration of the in vitro ADCP assay (A). Typical flow cytometry dot plots quantifying the proportion of effector cells that took up the HCV E2 protein‐coated microbeads and overlayed histograms showing mean florescence intensities (MFI) to measure the magnitude of bead uptake per cell (B). (I) A representative dot plot for HCV‐E2 protein‐coated nonopsonized fluorescent microbeads as background control; (II) HCV E2 protein‐coated fluorescent microbeads opsonized with healthy control plasma and; (III) HCV E2 protein‐coated fluorescent microbeads opsonized with a plasma of a patient with HCV (percentages of positive phagocytosis are shown on the upper and lower right quadrants of each plot). (IV) A typical histogram showing the MFI for HCV‐E2 protein‐coated nonopsonized fluorescent microbeads as a background control (orange) overlayed with a typical histogram for HCV E2 protein‐coated fluorescent microbeads opsonized with healthy control plasma (red) and a typical histogram for HCV E2 protein‐coated fluorescent microbeads opsonized with a plasma of a patient with HCV (blue).

THP‐1 cells (1 × 10^5^ cells in 50 μL PBS) were added onto 50–70 μL of the above opsonised microbeads, and volume was adjusted to 600 μL in Eppendorf tubes using RPMI containing 0.1% human serum albumin and 100 mM HEPES assay buffer. THP‐1 cells incubated with plain microbeads or nonopsonized microbeads coated with recombinant E2 protein were used as assay controls. Cell‐microbead mixtures were then incubated in a humidified 37°C, 5% CO_2_ chamber for 2 h, washed once with cold PBS + 0.5% FBS and 0.005% of sodium azide, fixed with 1% paraformaldehyde, and analyzed using the BD FACSCalibur™ flow cytometer.^61^ A total of 2 × 10^4^ events per tube were acquired from one sample tube from patients (*n* = 67) and healthy controls (*n* = 15). Relevant assay controls included the acquisition of 2 × 10^4^ events per tube from cells incubated with no beads and E2‐coated nonopsonized beads. The proportions of cells that phagocytosed the microbeads and mean fluorescence intensities (MFI) were analyzed using BD FlowJo version 10.5.0 software, and phagocytic scores (p‐score) were calculated as $\left(\frac{\%\;\mathrm{bead}\;\mathrm{positive}\;monocytes\times \mathrm{MFI}}{100}\right)\text{as}$ previously described.^28^, ^61^, ^66^ A positive p‐score was defined as three standard deviations above the background mean phagocytic score of healthy donors.^61^ A schematic diagram of the ADCP and representative data is shown in Figure 7.

### 4.8 Antibody‐mediated neutralization of HuH7.5 cells infection by E1E2 containing GT1a or GT3a HCV pseudovirus (HCVpp)

Envelope proteins 1 and 2 (E1E2) containing GT1a (H77) or GT3a (UKN3A13.6) HCVpp were generated in CD81 knockout 293T cells by co‐transfecting expression plasmids containing E1E2 and Murine Leukemia Virus (MLV) gag/pol and a chemiluminescent luciferase vector using Calphos Takara Bio transfection kit (Takara Bio) as described.^67^ Briefly, GT1a or GT3a HCVpp in culture supernatants from the transfected 293 T cells were treated with heat‐inactivated patient or healthy control sera in at 1:50 dilution for 1 h before infecting HuH7.5 cells by a 2‐h centrifugal inoculation in 96‐well flat‐bottom plates followed by a 2‐h incubation at 37°C and 5% CO_2_. After removing the excess HCVpp, the infected HuH7.5 cells were replenished with fresh media, incubated at 37°C and 5% CO_2_ for 72 h, and lysed with a lysis buffer (Promega). Relative Luminescence Unit (RLU) in cell lysates was measured using a CLARIOstar microplate reader at 450 nm, and percentages of antibody‐mediated neutralization were calculated as $\left(1-\frac{\mathrm{RLU}\;\mathrm{in}\;\mathrm{diluted}\;\mathrm{serum}}{\mathrm{RLU}\;\mathrm{no}\;\mathrm{serum}}\right)\times 100$. Positive neutralization was defined as a percentage larger than at least two standard deviations above the mean percentages obtained from 10 healthy donors. Negative neutralization values indicate enhancement of HCVpp infection upon incubation with patient serum as previously reported.^13^, ^33^

### 4.9 Surface plasmon resonance to characterize the binding of patient anti‐E2 antibodies to GT1a E2

Biacore T200 surface plasmon resonance (SPR) (Cytiva) was used to determine the interaction of anti‐E2 antibodies in patient plasma with recombinant E2 protein in real time. Briefly, 10 µg/mL recombinant E2 containing a C‐terminal 6x‐His‐tag was captured by an anti‐His‐tag monoclonal antibody that was immobilized onto carboxymethylated (CM5) dextran sensor chips at a flow rate of 10 µL per minutes for 420 s using EDC/NHS amine coupling kit (GE Healthcare). The sensor chips were then equilibrated with a pH 7.4 running buffer containing HBS‐EP +, 0.01 M HEPES, 150 mM NaCl, 3 mM EDTA and 0.05% v/v Surfactant P20 and plasma from patients or healthy controls diluted 1:100 in the running buffer was injected to the flow cells of the Biacore T200 at a rate of 20 µL per minutes for 120 s at 25°C to determine how fast the anti‐E2‐E2 bind to each other (association constant; *k*_*a*_), how fast the complexes come apart (dissociation constant; *k*_*d*_) and how strong is the interaction between the antibodies and E2 (affinity; *K*_*D*_).^68^ Nonspecific binding of plasma to an empty flow cell and from a flow cell with a blank injection (zero analyte concentration) were used as background controls. After each run, chips were washed with PBS, pH 7.4, regenerated using 10 mM glycine, pH 2.0, equilibrated with running buffer and reused.

### 4.10 Mapping of epitopes on the GT1a E2 protein targeted by antibodies in patient plasma

An epitope is a certain sequence of amino acids on a pathogen that allows for specific binding of a given antibody. In HCV infection, several neutralizing antibodies have been identified to target specific epitopes on certain antigenic regions or domains of the E2 protein (Antigenic Regions 1–5; also known as Domains A–E or Epitopes I–III), although some are found to target conformational epitopes made up of both E1 and E2 residues.^30^ Importantly, these antibodies vary in their neutralization potency and breath,^58^, ^69^ however, whether they vary in their ability to provoke ADCP and if levels of ADCP correlate with antibodies that target certain epitopes remain unknown. To address this, HCV E2 epitopes targeted by antibodies in the plasma of patients infected with GT1a virus were mapped by competitive ELISA using monoclonal antibodies that bind specific epitopes on E2 protein and epitope mapping scores (EMS) determined as described.^58^ The relationship between the anti‐E2 antibodies in patient plasma targeting the different epitopes of E2 was then correlated with their phagocytic and neutralization function. The competitive monoclonal antibodies used were clones AR1B, AR2A, AR3A, AR4A, AR5A (donated by Prof. Mansun Law, The Scripps Research Institute, La Jolla, USA), HC84.26 provided by Prof. Steven Foung, Stanford University, USA), CBH4G, and CBH7 purified in‐house from supernatants of PTA‐4468 and PTA‐4470 hybridoma from ATCC and HCV1 produced by transient transfection of 293F cells and biotinylated using EZ‐Link® Sulfo‐NHS‐LC‐Biotin kit.^58^

### 4.11 Statistical analysis

All data were analyzed using Prism Software (version 9.0, GraphPad). Unpaired nonparametric Mann–Whitney *U*‐test was used to compare anti‐E2 antibody levels in plasma, phagocytic scores (E2 p‐scores), and ID_50_ of the antibody‐mediated neutralization between patients with chronic HCV and those who cleared the virus. When there were more than two groups, analysis of variance with Dunn's test for correction or the Kruskal–Wallis test was used. Spearman's correlation coefficient was used to correlate levels of anti‐E2 IgG to E2 p‐scores, percentages of antibody‐mediated neutralization or affinity. Statistical parameter correlation matrices were used to assess whether antibodies in patient plasma targeting the different E2 epitopes have a positive or negative relationship to ADCP or neutralization functions and comparison of their relative significance were plotted on covariance matrices and associative relationships among the independent variables (*r* = Spearman correlation) were determined where values greater than zero are considered a positive relationship, zero represented no relationship, and values less than 1 indicated a negative.

## AUTHOR CONTRIBUTIONS

Anurag Adhikari, Nicodemus Tedla, and Rowena Bull: Conceptualization and project administration. Anurag Adhikari, Nicodemus Tedla, Rowena Bull, Arunasingam Abayasingam, Nicholas A. Brasher, Ha Na Kim, Megan Lord, Chaturaka Rodrigo, David Agapiou, and Andrew R. Lloyd: Data curation; formal analysis; methodology; and investigation. Nicodemus Tedla, Rowena Bull, Lisa Maher, and Andrew Lloyd: Funding acquisition and resources. Nicodemus Tedla, Chaturaka Rodrigo, and Rowena Bull: Supervision. Anurag Adhikari: Writing of original draft. Nicodemus Tedla, Rowena Bull, Chaturaka Rodrigo, and Andrew Lloyd: Reviewing and editing.

## CONFLICT OF INTEREST STATEMENT

The authors declare no conflict of interest.

## Supporting information


**Supplementary Figure 1: Non‐significant sex‐dependent differences in anti‐E2 antibody levels, ADCP, and neutralisation functions**. (a) Difference in the concentration of plasma anti‐E2 antibodies between males (mean = 108.2 ± 34.8 µg mL‐1) and females (mean = 80 ± 14.0 µg mL‐1), and (b) the difference in phagocytic function between male and female patients (mean = 185.9 ± 51.8 versus 142.7 ± 37.5; P = ns), (c) and the difference in neutralization function between male and female patients (mean = 39.1 ± 5.8% versus 30.8 ± 4.9%; P = ns). (d, e, f) Regardless of the infecting viral genotype, there were no significant differences observed between males and females in terms of (d) anti‐E2 antibody concentration, (e) phagocytosis, or (f) neutralization.

**Supplementary Figure 2: Epitope mapping scores of antibodies in plasma of patients with GT1a HCV infection**. Antibodies targeting Domain B (AR2A and AR3A) and those targeting Domain D (HC84.26) that were positively associated with high ADCP and neutralisation function were also relatively higher than those representing Domain C (CBH‐7), Domain E (HCV‐1) and Domains A that showed negative association. These results were particularly evident in patients with chronic disease, likely due to the presence of high titres of total anti‐E2 antibodies in these patients compared to those who cleared the virus.

## Data Availability

The data that support the findings of this study are available from the corresponding author upon reasonable request.
