# Supplementary figures and images for: Characterization of antibody‐dependent cellular phagocytosis in patients infected with hepatitis C virus with different clinical outcomes
Source: J Med Virol. 2024 Jan 18;96(1):e29381. doi: 10.1002/jmv.29381 (PMC10953302; doi:10.1002/jmv.29381)

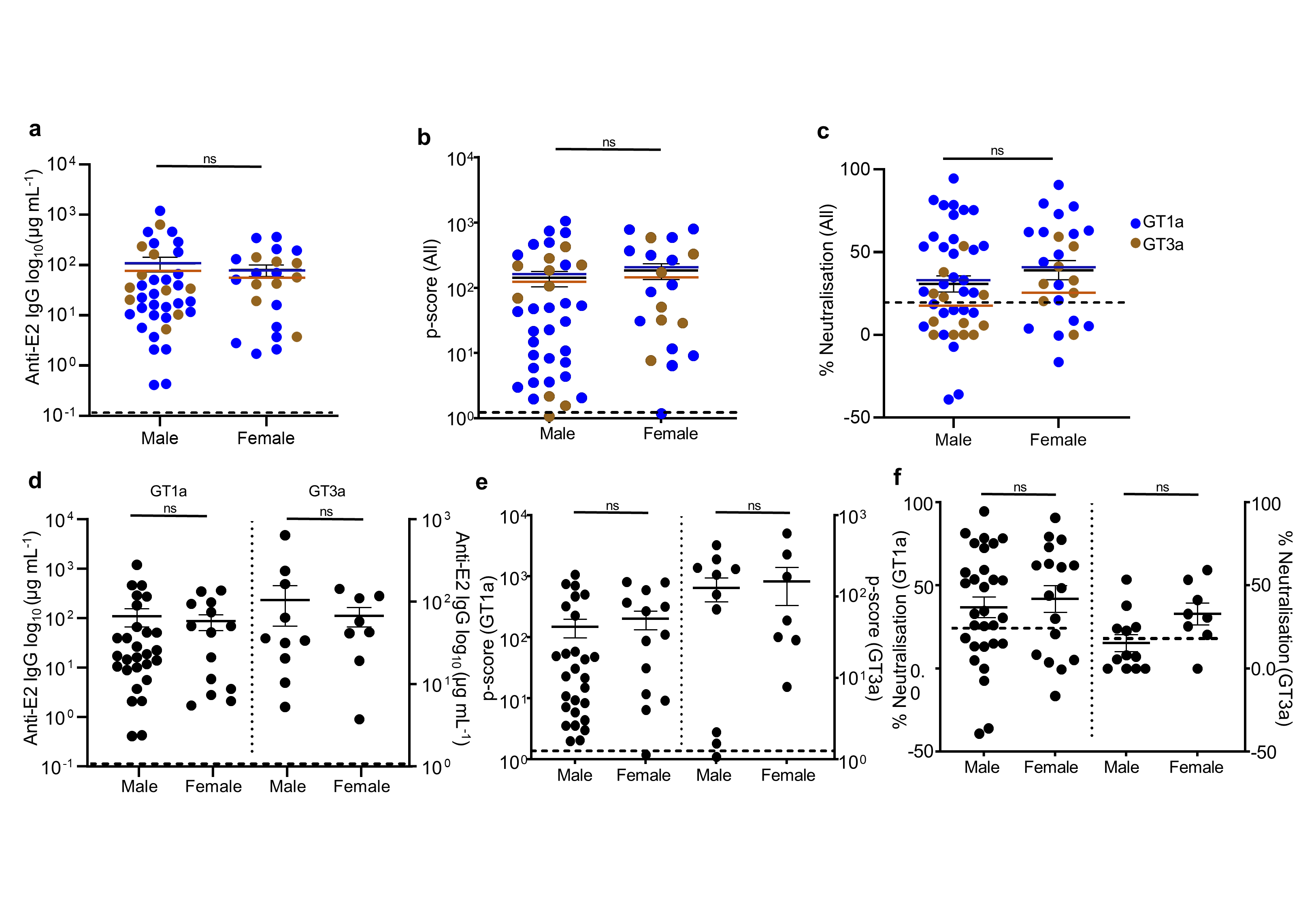

Supplement: Supplementary file 1 — Supplementary Figure 1: Non‐significant sex‐dependent differences in anti‐E2 antibody levels, ADCP, and neutralisation functions. (a) Difference in the concentration of plasma anti‐E2 antibodies between males (mean = 108.2 ± 34.8 µg mL‐1) and females (mean = 80 ± 14.0 µg mL‐1), and (b) the difference in phagocytic function between male and female patients (mean = 185.9 ± 51.8 versus 142.7 ± 37.5; P = ns), (c) and the difference in neutralization function between male and female patients (mean = 39.1 ± 5.8% versus 30.8 ± 4.9%; P = ns). (d, e, f) Regardless of the infecting viral genotype, there were no significant differences observed between males and females in terms of (d) anti‐E2 antibody concentration, (e) phagocytosis, or (f) neutralization. [file JMV-96-0-s002.tif]

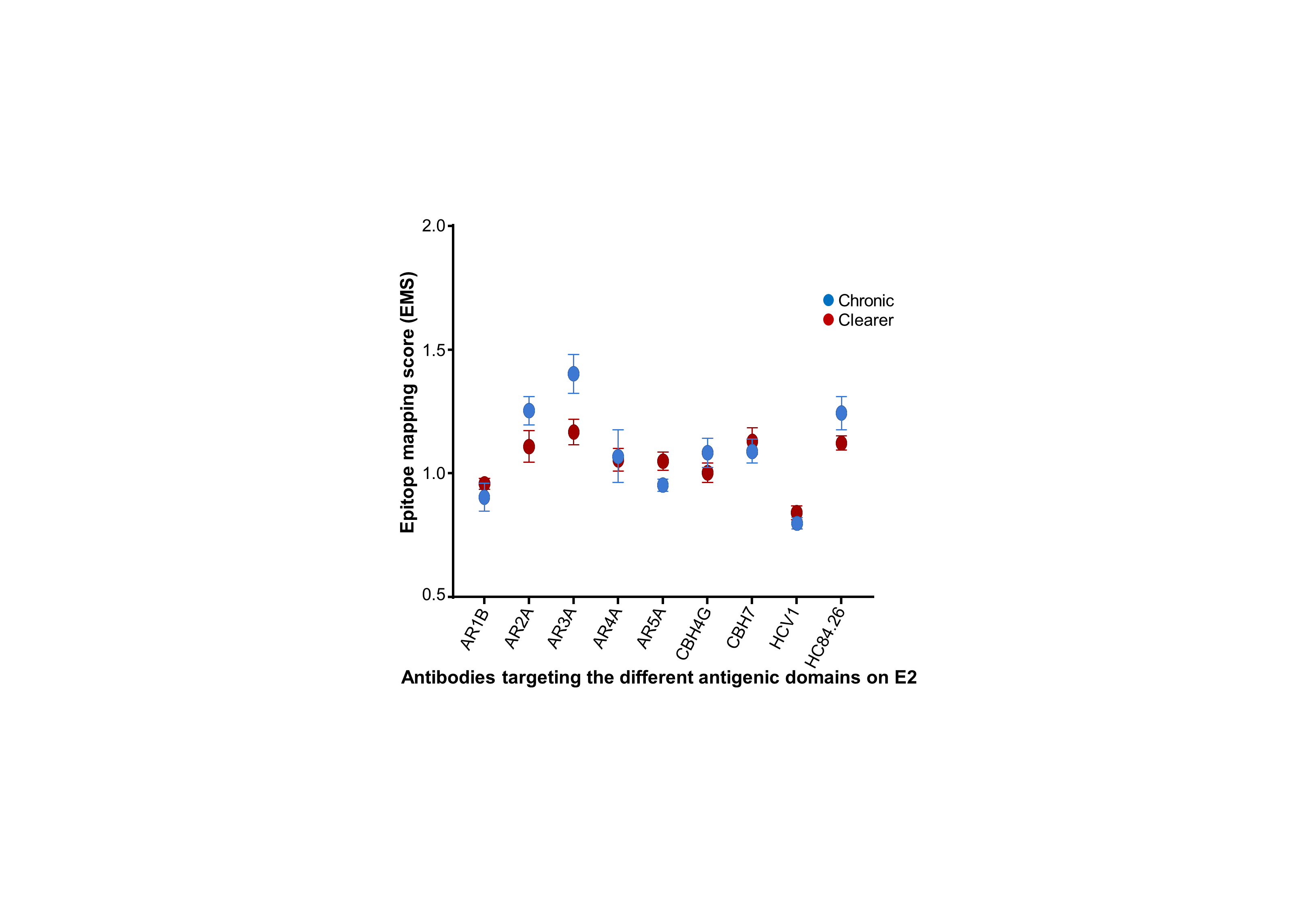

Supplement: Supplementary file 2 — Supplementary Figure 2: Epitope mapping scores of antibodies in plasma of patients with GT1a HCV infection. Antibodies targeting Domain B (AR2A and AR3A) and those targeting Domain D (HC84.26) that were positively associated with high ADCP and neutralisation function were also relatively higher than those representing Domain C (CBH‐7), Domain E (HCV‐1) and Domains A that showed negative association. These results were particularly evident in patients with chronic disease, likely due to the presence of high titres of total anti‐E2 antibodies in these patients compared to those who cleared the virus. [file JMV-96-0-s001.tif]
